# Telerehabilitation robotics for upper limb rehabilitation after stroke (TRUST): a multi-site pragmatic trial protocol

**DOI:** 10.3389/fneur.2026.1775829

**Published:** 2026-05-21

**Authors:** Ravi Shankar, Silvana Xinyi Choo, Zhenzhen Chen, Christopher Wee Keong Kuah, Tegan Kate Plunkett, Chwee Yin Ng, Sijie Lin, Kim Huat Goh, Emily Yee, Xiaojia Ge, Doris Zhang, Wei Binh Chong, Jaclyn Ai Mei Low, Megan Si En Lau, Xin Yi Lim, Saung Yupar Naing, Lian Ting Wong, Bernardo Noronha, Gabriel Aguirre-Ollinger, Asif Hussain, Poo Lee Ong, Karen Sui Geok Chua

**Affiliations:** 1 Clinical Research and Innovation Office, Tan Tock Seng Hospital, National Healthcare Group Health, Singapore, Singapore; 2 Department of Occupational Therapy, Singapore General Hospital, Singapore, Singapore; 3 Health & Social Sciences Cluster, Singapore Institute of Technology, Singapore, Singapore; 4 Department of Rehabilitation, National University Hospital, Singapore, Singapore; 5 Institute of Rehabilitation Excellence (IREx), Tan Tock Seng Hospital Rehabilitation Centre, National Healthcare Group Health, Singapore, Singapore; 6 Nanyang Business School, Nanyang Technological University, Singapore, Singapore; 7 Articares Pte Ltd., Singapore, Singapore; 8 Rehabilitation Research Institute of Singapore (RRIS), Lee Kong Chian School of Medicine, Nanyang Technological University, Singapore, Singapore; 9 Yong Loo Lin School of Medicine, National University of Singapore, Singapore, Singapore

**Keywords:** rehabilitation, robotics, stroke, telerehabilitation, upper limb

## Abstract

**Background:**

Stroke remains a leading cause of long-term disability globally, with upper limb impairment persisting in 39% of survivors at six months. The post-acute rehabilitation gap, characterized by precipitous decline in therapy intensity after hospital discharge, compromises recovery during critical neuroplastic windows. While robot-aided therapy (RAT) demonstrates efficacy comparable to conventional therapy, widespread implementation remains limited by cost, complexity, and facility-based care models. Home-based, minimally supervised RAT with telemonitoring could address these barriers while providing intensive therapy without proportional increases in healthcare resources.

**Objective:**

To evaluate the feasibility, acceptability, clinical effectiveness, and cost-effectiveness of home-based minimally supervised RAT using a portable planar robot in patients with moderate upper limb impairment following stroke.

**Methods:**

This pragmatic, single-arm, multi-center prospective trial will recruit 54 adults with subacute to chronic stroke (>28 days post-event) with upper extremity impairment (Fugl-Meyer Assessment 10–60) across three tertiary rehabilitation centers in Singapore. The intervention comprises four weeks of intensive home-based, caregiver-supervised training using the H-Man robot (60–120 min daily) with weekly telemonitoring, combined with four conventional occupational therapy sessions over an eight-week treatment period. The primary outcome is utilization rate (≥75% compliance) measured by cloud-based metrics. Secondary outcomes include motor impairment (FMA-UE), functional capacity (ARAT), quality of life (EQ-5D-5L), usability (System Usability Scale), and technology acceptance, assessed at baseline, 6, 8, 12, and 24 weeks. Economic evaluation will assess cost-effectiveness from healthcare system and societal perspectives.

**Results:**

Recruitment commenced October 2024 across three Singapore rehabilitation hospitals. The study employs rigorous safety monitoring, standardized training protocols, and cloud-based performance tracking. Preliminary feasibility testing demonstrates acceptable device usability and caregiver support requirements.

**Conclusion:**

TRUST addresses critical gaps in post-stroke rehabilitation by evaluating a scalable model combining technology-enabled home therapy with remote clinical oversight. Results will inform implementation strategies for integrating home-RAT into standard care pathways, potentially transforming stroke rehabilitation delivery in resource-constrained healthcare systems facing growing demand from aging populations.

**Clinical trial registration:**

ClinicalTrials.gov, identifier NCT06270459.

## 1 Introduction

### 1.1 The burden of stroke and upper limb dysfunction

Stroke represents a major global health challenge, ranking as the second leading cause of death with 6.2 m worldwide, accounting for 11% of all deaths; and the third leading cause of disability-adjusted life years lost worldwide (1). The societal impact extends far beyond acute mortality, with survivors experiencing persistent functional impairments that profoundly affect independence, quality of life, and caregiver burden. Upper extremity (UE) motor impairment emerges as one of the most prevalent and disabling sequelae, persisting in approximately 39% of stroke survivors at six months post-event (2). This persistent dysfunction significantly limits activities of daily living, vocational participation, and social engagement, creating cascading effects on psychological wellbeing and family dynamics.

In Singapore, stroke is the 4th leading cause of death, accounting for 5.6% of all deaths and affecting 9.3% of adults over 65 years (3). The burden of stroke is projected to increase substantially by 2030 as a consequence of rapid population aging in Singapore and its official status in 2026 as a superaged society (3). The age-adjusted prevalence of ischemic stroke reaches 125.06 per 100,000 person-years, with hemorrhagic stroke at 44.19 per 100,000 person-years (4). Stroke rehabilitation is a lifeline of hope for stroke survivors and caregivers from early days to years’ post-stroke as it reduces stroke-related complications, dependency, disability, institutionalization and increase functional independence and societal reintegration. These demographics underscore the urgent need for scalable, effective rehabilitation interventions that can meet growing demand while maintaining quality within resource constraints.

### 1.2 The post-acute rehabilitation gap

Critical and chronic discontinuity characterizes the local stroke rehabilitation continuum, manifesting as a precipitous decline in therapy intensity following hospital discharge. During inpatient rehabilitation, patients typically receive intensive daily therapy (3h, 3H/day) aligned with evidence-based recommendations for optimizing neuroplastic recovery. However, this intensity drops dramatically post-discharge, with first outpatient contact varying from 2–6 weeks and subsequent therapy frequency ranging from biweekly to monthly sessions (5). This “rehabilitation cliff effect” causes a major systemic mismatch for the critical neuroplastic window when recovery potential remains high but vulnerability to deterioration peaks.

The consequences of inadequate post-acute rehabilitation extend beyond delayed recovery. Learned non-use, characterized by progressive abandonment of the use of the affected limb despite residual function, develops through repeated failure experiences and compensatory strategies (6). Hidaka et al. (7) demonstrated through computational modeling that the bidirectional relationship between arm function and use creates feedback loops—increased use promotes functional improvement, while decreased use accelerates decline. These dynamics underscore the critical importance of maintaining therapy intensity during the post-acute phase.

### 1.3 Robot-aided therapy: evidence and implementation barriers

Robot-aided therapy has emerged as an evidence-based approach to deliver high-intensity, task-specific rehabilitation addressing fundamental challenges in stroke care. The mechanistic basis for RAT effectiveness lies in enabling high-dose repetitive practice that promotes use-dependent plasticity while providing precise kinematic and kinetic measurement for objective progress monitoring (8). Multiple systematic reviews and meta-analyses demonstrate that RAT achieves outcomes comparable to dose-matched conventional therapy for motor impairment reduction, with potential advantages when used as an adjunct rather than replacement (9, 10).

Despite robust evidence, RAT implementation remains severely limited. Commercial systems cost SGD 100,000–500,000, require dedicated space and technical support, and lack reimbursement coverage in many healthcare systems. These barriers confine RAT primarily to well-resourced rehabilitation centers, perpetuating access inequities. The COVID-19 pandemic further exposed vulnerabilities in facility-based models, with service disruptions disproportionately affecting stroke survivors requiring consistent intensive therapy (11).

This manuscript describes a pragmatic trial protocol rather than a systematic review; accordingly, no formal systematic search strategy was employed. The background evidence summarized in the Introduction is drawn from landmark systematic reviews, meta-analyses, and seminal trials in robot-aided stroke rehabilitation identified through targeted literature review of key databases (PubMed, Cochrane Library) and citation tracking of foundational works in the field. A comprehensive list of supporting references is provided.

### 1.4 The H-Man system: democratizing access to robotic rehabilitation

The H-Man robot, developed through collaboration between Nanyang Technological University and Tan Tock Seng Hospital, represents a paradigm shift in rehabilitation robotics design philosophy. Prioritizing accessibility over complexity, H-Man achieved CE Mark certification as a Class 2A medical device in 2020 for hospital, clinic, and critically, home use. At approximately 25% the cost of commercial alternatives, weighing 14 kg, and requiring only 880 × 700 mm table space, H-Man addresses key implementation barriers (12).

Technical specifications reflect careful balance between functionality and simplicity. The planar manipulandum with two active degrees of freedom enables reaching and drawing movements fundamental to upper limb function. The H-shape cabled differential transmission provides intrinsic safety through mechanical compliance, eliminating complex force-limiting software that increases cost and reduces reliability. A previous randomized controlled trial demonstrated that H-Man-assisted therapy combined with conventional therapy achieved motor improvements statistically equivalent to duration-matched conventional therapy alone in 44 chronic stroke survivors, with no serious adverse events and high satisfaction scores (13, 15).

### 1.5 Study rationale and objectives

Current care models in Singapore’s public healthcare institutions cannot provide daily intensive rehabilitation without substantially increasing resource burden. The shift from facility-centric to home-based care using affordable, portable robotic technology could ensure therapy continuity while addressing multiple access barriers. This pragmatic trial evaluates real-world implementation of home-RAT across diverse healthcare settings, prioritizing generalizability and inclusivity over experimental control.

The primary objective is to determine the utilization rate of home-based RAT, defined as the proportion of prescribed sessions completed (≥75% threshold), as this directly informs implementation viability. Secondary objectives include: (1) establishing feasibility and acceptability of home-RAT implementation across multiple sites; (2) evaluating clinical effectiveness on motor and functional outcomes; (3) assessing economic viability from healthcare system and societal perspectives; and (4) identifying implementation facilitators and barriers to inform scale-up strategies.

## 2 Methods

### 2.1 Study design and setting

This pragmatic, single-arm, multi-site prospective trial employs an implementation-effective hybrid design (Type 2) prioritizing both clinical outcomes and implementation factors (14). The single-arm design was selected over randomized controlled trial designs based on the presence of: (1) extensive existing evidence for RAT equivalent efficacy in comparison with usual care; (2) ethical considerations about withholding potentially beneficial intervention with a randomized control design employing a control group; (3) practical challenges of standardizing rehabilitation treatment control conditions across sites; and (4) the pressing need to evaluate the feasibility, QOL and cost-effectiveness of delivering home-RAT rather than efficacy under ideal or standardised or highly supervised home-training conditions.

The study is conducted across three major public healthcare institutions in Singapore:

- Tan Tock Seng Hospital (TTSH), National Healthcare Group Health (NHG health): Lead site with Singapore’s first and largest rehabilitation medicine department with extensive rehabilitation technology clinical practice and research experience.
- Singapore General Hospital (SGH), Singapore Health Services (Singhealth): Nation’s largest hospital with integrated acute-subacute-outpatient rehabilitation services
- National University Hospital (NUH), National University Health System (NUHS): Academic medical center with Integrated academic health system with strong translational research infrastructure

Each site serves demographically and clinically distinct populations. TTSH predominantly manages older adults with higher neurological complexity through its specialized rehabilitation centre, SGH serves a broad acute-to-chronic case mix through its integrated rehabilitation pathway, and NUH draws from its academic health system catchment with a higher proportion of younger stroke survivors and research-naïve participants. These sites employ different care models, enhancing generalizability while testing implementation robustness across contexts.

### 2.2 Participants

#### 2.2.1 Eligibility criteria

Several eligibility criteria warrant clarification regarding operationalization. ‘Near-normal function’ is defined as FMA-UE > 60, indicating minimal residual motor impairment insufficient to benefit meaningfully from robotic-assisted training. ‘Medical stability’ is determined by the referring rehabilitation physician based on the absence of acute intercurrent illness, haemodynamic instability, or uncontrolled comorbidities requiring active medical titration within the preceding two weeks. ‘mild cognitive impairment’ is operationalized as a Montreal Cognitive Assessment (MoCA) score ≤21, consistent with the cognitive inclusion threshold. All eligibility determinations are documented by the site principal investigator using a standardized screening checklist. Participants with mild shoulder subluxation (≤1 finger-breadth) or controlled pain (VAS ≤ 5/10) and, pain is monitored longitudinally using the Visual Analogue Scale at all assessment timepoints (see Table 1).

**Table 1:** Inclusion and exclusion criteria.

Domain	Inclusion criteria	Exclusion criteria
Diagnosis	Confirmed ischemic or hemorrhagic stroke Neuroimaging confirmation by neurologists or neurosurgeons	Non-stroke etiology of impairment Stroke impairment mimics
Demographics	Age 21–90 years Both genders	Pregnancy or lactation
Timing	>28 days post-stroke	Life expectancy <6 months
Motor Function	MRC ≥ 2/5 shoulder/elbow FMA 10–60	Complete paralysis Near-normal function
Cognitive	MOCA >21/30 Ability to consent/assent and understand demands of study	Mild cognitive impairment Active psychiatric illness
Physical	Sitting tolerance >60 min Medical stability	Orthostatic hypotension Active seizures Medical instability Severe shoulder subluxation (≥2 finger-breadths) with pain at rest Uncontrolled upper limb pain (VAS > 5/10 at rest) unresponsive to analgesia
Environmental	Stable home during trial duration Available caregiver	Homeless or unstable housing Absence of reliable caregiver /No support system/Resident of nursing home
Medical	Controlled comorbidities	Uncontrolled medical illness Active cancer

#### 2.2.2 Sample size calculation

Sample size determination used non-inferiority framework with utilization rate as primary outcome. Based on 70% utilization in clinic-based programs (historical controls) and 5% non-inferiority margin, 18 participants per site (*n* = 54 total) provides 80% power at *α* = 0.10 (one-sided). This sample accounts for an anticipated 15% dropout rate (approximately 8 participants), yielding a minimum evaluable sample of 46 participants. Dropout is defined as withdrawal of consent, loss to follow-up, or inability to complete ≥50% of the home-RAT phase. Sensitivity analyses will compare intention-to-treat (all enrolled) and per-protocol (≥75% session completion) populations.

### 2.3 Intervention protocol

**Figure 1:**
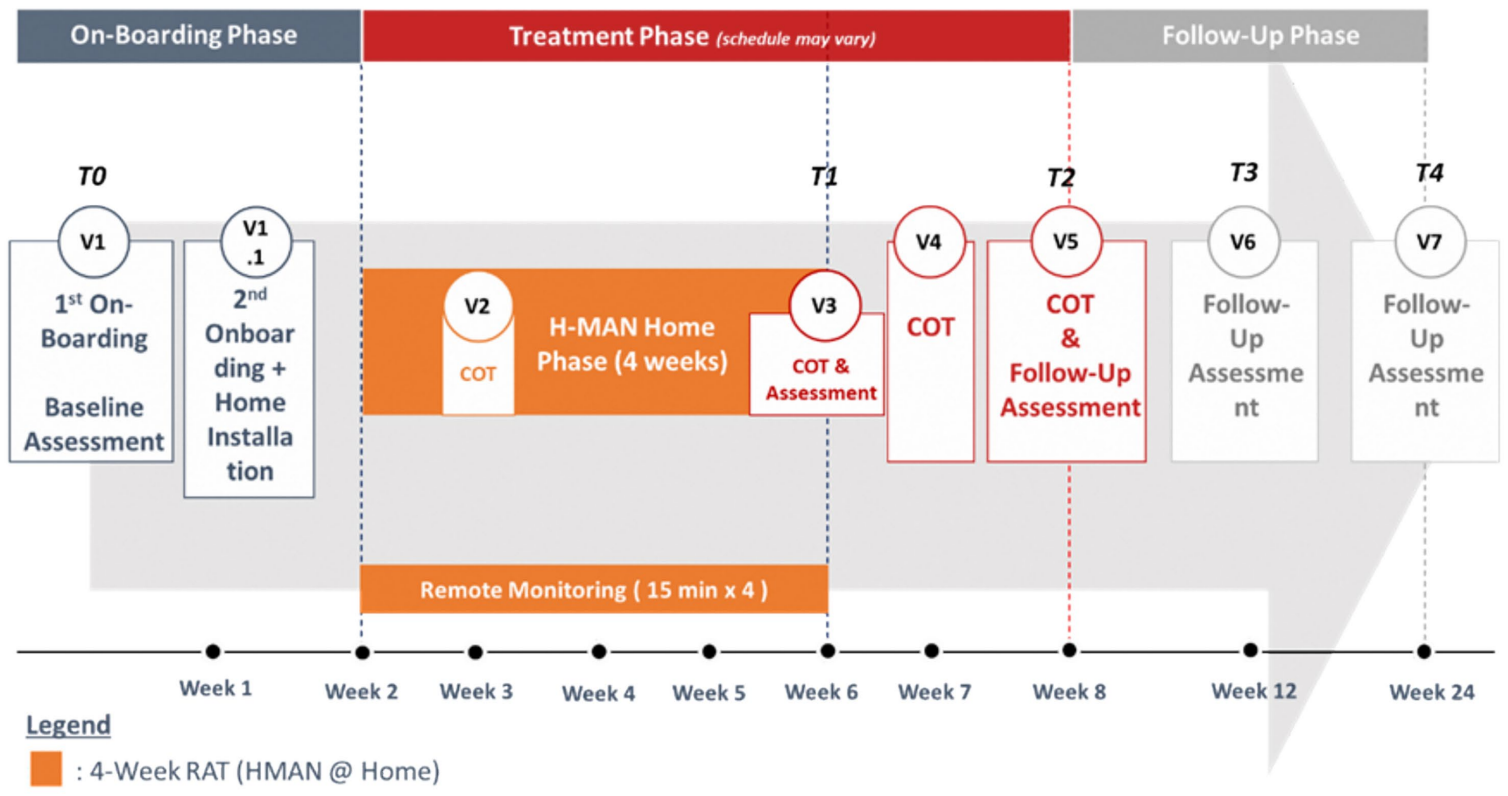
Study protocol.

The intervention integrates home-RAT with scheduled clinic visits across 3 phases over 24 weeks including an 8-week interventions phase and 16-week follow-up phase. Written informed consent will be taken from all eligible participants prior to enrolment. Refer to Figure 1 for the study protocol for all 3 hospitals.

#### 2.3.1 Phase 1: onboarding and installation (week 0)

- Visit 1 (clinic): baseline assessments, initial H-Man orientation and training, supervised 1:1 by rehabilitation therapist (90 min)
- Visit 1.1 (home): device installation by industry vendor, H-Man set up by rehabilitation therapist, programme setting and caregiver training on H-Man operations, safe mode of operation, troubleshooting with company FAQs (120 min)

#### 2.3.2 Phase 2: treatment (weeks 1–8)

- Home training (weeks 1–4, total 28 days): daily self-directed H-Man therapy, gradually increasing from 30 min per day during the first three days to 60 min by Day 4, and up to 120 min (with mandatory rest breaks every 30 min) from Day 7 onward, based on a standardized progression protocol. Escalation decisions are guided by predefined criteria: absence of increased pain (VAS increase ≤2 points), fatigue rated ≤6/10 on a self-reported scale, and no adverse events in the preceding sessions. The treating therapist reviews cloud-uploaded performance data weekly during telemonitoring calls and may recommend maintaining, increasing, or reducing duration. Participants are instructed not to exceed 120 min per day, caregiver supervision as needed. Participants were encouraged to increase training duration by repeating the prescribed games as tolerated, if no adverse effects such as arm pain or fatigue or increased muscle tone were noted. Similarly, participants had the option to reduce training duration per session/daily episodes or take breaks when needed. Therapists could remotely adjust or modify prescribed gameplay exercises or difficulty. Weekly remote monitoring was supplemented by phone calls as needed and in person checks during COT sessions.
- Telemonitoring: weekly 15-min therapist review of cloud-uploaded performance data with phone consultations if non-compliance or overexercise or variable performance are noted.
- Conventional therapy: four 60-min sessions at fortnightly intervals, conducted by rehabilitation therapist, during 8 weeks (2 sessions during home-RAT phase and 2 sessions after the home-RAT phase)
- Assessments: mid-treatment post-home-RAT (Week 6) and post-COT/end-treatment (Week 8) by occupational therapists or trained assessors.

#### 2.3.3 Phase 3: follow-up (weeks 9–24)

- Assessments: week 12 and 24 to evaluate sustainability of gains

All formal outcome assessments (baseline, Week 6, Week 8, Week 12, and Week 24) are conducted in-clinic at the respective study sites by the designated assessor, ensuring standardized testing conditions and access to calibrated equipment (e.g., Jamar dynamometer). The in-clinic setting also facilitates clinical safety review at each assessment timepoint (see Table 2).

**Table 2:** Intervention schedule and assessment timeline.

Week	0	1–4	6	8	12	24
Phase	Onboarding	Home Training	Mid-Treatment	End-Treatment	Follow-up	Follow-up
H-MAN Training	Training	Daily 60–120 min	Continued	Complete	-	-
Conventional occupational Therapy (COT)	-	2 sessions, fortnightly intervals	1 session	1 session	-	-
Telemonitoring	-	Weekly x4	-	-	-	-
Assessments (total 5)	Baseline	-	Primary	Secondary	Maintenance	Long-term

To ensure consistency and minimize bias, each site designates a primary treating therapist responsible for all enrolled participants at that site, including onboarding, telemonitoring, and conventional therapy sessions. Outcome assessments are conducted by a separate trained assessor at each site who is not involved in treatment delivery, thereby maintaining blinding to treatment progress. All assessors undergo centralized training using standardized assessment protocols and video calibration sessions prior to study commencement, with inter-rater reliability established at >0.85 (ICC) for FMA-UE and ARAT.

### 2.4 H-Man system specifications

The H-Man robot’s design philosophy prioritizes clinical functionality and home deployment feasibility over technological complexity, achieving a balance that addresses key implementation barriers while maintaining therapeutic effectiveness. Technical specifications were optimized through iterative development with stroke survivors and therapists, ensuring the system meets clinical requirements for motor relearning while remaining accessible for home use. The device’s compact form factor, intrinsic safety features, and simplified operation enable deployment in diverse home environments without specialized infrastructure or technical expertise. Carers can be trained for independent operations of H-Man, and Wi-Fi is not essential for operations. Data upload to cloud occurs with adequate Wi-Fi bandwidth.

Table 3 presents detailed technical parameters alongside their clinical relevance for stroke rehabilitation.

**Table 3:** H-MAN technical specifications.

Parameter	Specification	Clinical Relevance
Physical		
Weight	14 kg	Caregiver portable
Dimensions	880 × 700 × 400 mm	Standard table compatible
Workspace	400 × 300 mm planar	Functional reach range
Mechanical		
Degrees of Freedom	2 active (x,y)	Planar reaching/drawing
Transmission	H-cable differential	Intrinsic safety
Force Capacity	50 N maximum	Therapeutic resistance
Control		
Sampling Rate	1,000 Hz	Smooth interaction
Impedance Control	0–50 N/m adjustable	Progressive assistance
Interface		
Display	15″ touchscreen	Clear visualization
Games	8 VR environments	Varied practice
Feedback	Visual + haptic + audio	Multimodal learning
Data		
Storage	32GB local + cloud	Offline capability
Connectivity	Wi-Fi (intermittent OK)	Flexible deployment

The H-Man robot is manufactured and distributed by Articares Pte Ltd. (Singapore), which provides device installation, technical maintenance, and replacement units under warranty this interests statement.

#### 2.4.1 H-Man training protocol and therapeutic content

The H-Man training protocol comprises structured exergaming activities designed to promote repetitive, goal-directed upper limb movements within the planar workspace. Training targets three primary movement patterns: (a) point-to-point reaching in multiple directions, training shoulder flexion-extension and abduction-adduction with elbow coordination; (b) continuous tracing and drawing tasks requiring sustained smooth movement control; and (c) functional trajectory movements simulating activities such as wiping and reaching-to-grasp patterns.

The H-Man system provides three modes of robot-patient interaction: (i) active-assisted mode, where the robot supplements patient-initiated movement toward targets with adjustable impedance (0–50 N/m); (ii) active-resisted mode, providing resistance against patient movement to strengthen motor output; and (iii) free active mode, where the robot provides no assistance or resistance and serves only as a measurement instrument. Mode selection is prescribed by the treating therapist during onboarding based on baseline motor capacity and is adjustable during weekly telemonitoring reviews.

The exergaming interface offers eight virtual reality game environments displayed on the integrated 15-inch touchscreen, including target-hitting, path-following, maze navigation, and object-sorting tasks. Each game provides multimodal feedback: visual feedback (real-time cursor tracking, target highlighting, and score display), haptic feedback (force cues from the robot handle indicating target proximity and movement errors), and auditory feedback (tones for successful target acquisition and verbal encouragement). Game difficulty auto-adjusts based on performance metrics including movement accuracy, speed, and smoothness. The treating therapist may recommend specific games and interaction modes during telemonitoring to target individual impairment profiles (e.g., emphasizing reaching accuracy for patients with dysmetria, or resistance training for those with proximal weakness). Training is thus individualized within a standardized framework rather than applied as a uniform protocol.

### 2.5 Outcome measures

#### 2.5.1 Primary outcome

##### 2.5.1.1 Utilization rate

Percentage of prescribed sessions completed (≥30 min/day active exergaming therapy without idling time was deemed as compliance/day), automatically captured via cloud monitoring. Success threshold ≥75% based on dose–response literature and based on prior validation trial for hospital-home-RAT.

#### 2.5.2 Secondary outcomes

##### 2.5.2.1 Motor and functional

- Upper extremity (UE) Fugl-Meyer Motor Assessment (FMA) is a widely used quantitative measure of motor impairment to evaluate upper-limb recovery. Its scores range from 0 being the minimum to the maximum score of 66 points and is divided into UE including shoulder-elbow, and coordination and speed (0–42) and distal wrist-hand scores (0–24).
- Action Research Arm Test (ARAT) is a 19-item observational measure of upper-extremity performance score and score ranges from 0 being the minimum to the maximum score of 57 points. It consists of 4 sub-tests (grasp., grip, pinch, and gross movement). Each task performance is rated on a 4-point scale ranging from 0 (no movement) to 3 (normal movement). The subscale ranges for each subtest are grasp (6 items, 0–18), grip (4 items, 0–12), pinch (6 items, 0–18) and gross movement (3 items, 0–9). Scores from each task are summed, with a minimum total score of 0 to a maximum score of 57.
- Affected hand grip strength is measured using Jamar Dynamometer (kg) using the mean reading of 3 attempts.
- Clinical measures of hemiparetic limb spasticity of shoulder adductors, elbow flexors, wrist and finger flexors using the Modified Ashworth Scale scores (MAS)
- Visual Analogue Scale: Pain assessment of shoulder and arm (0–10)

##### 2.5.2.2 Quality of life and participation

- EQ-5D-5L: health utility for economic analysis
- PROMIS-10: global health status
- Stroke impact scale-16: stroke-specific quality of life

##### 2.5.2.3 Technology acceptance

- System usability scale: perceived usability (0–100, >68 acceptable)
- Intrinsic motivation inventory: engagement and perceived value
- Technology acceptance model: intention to continue use

##### 2.5.2.4 Economic

- Direct medical costs: device, therapy time, clinic visits
- Direct non-medical costs: transportation, caregiver time
- Indirect costs: productivity losses
- Willingness-to-pay: contingent valuation

### 2.6 Data collection and management

The study employs multi-modal data collection through standardized clinical assessments by trained assessors (inter-rater reliability >0.85), secure REDCap database with validation rules and audit trails, automated cloud upload of robotic performance data (100 Hz sampling, ~50 MB/session), and structured medical record abstraction with double data entry for critical variables. Comprehensive data security includes AES-256 encryption for all transmissions, Singapore-based servers for data residency compliance, role-based access with two-factor authentication, and complete participant de-identification with separate storage of linking files. The H-Man system contains no identifiers, using only study IDs, with all data retained for 6 years per institutional requirements before secure destruction.

### 2.7 Statistical analysis plan

The primary analysis evaluates non-inferiority of the observed utilization rate against a 70% historical benchmark derived from clinic-based RAT programs, with a pre-specified non-inferiority margin of 5%. Non-inferiority is demonstrated if the lower bound of the one-sided 95% confidence interval for the observed utilization rate exceeds 65%. This analysis follows intention-to-treat principles, including all enrolled participants regardless of completion status; participants who withdraw are assigned zero utilization for remaining sessions.

For secondary clinical outcomes (FMA-UE, ARAT, grip strength, EQ-5D-5L, SIS-16, PROMIS-10), linear mixed-effects models are employed with fixed effects for time (categorical: baseline, Week 6, Week 8, Week 12, Week 24), site, and baseline severity (FMA-UE score), and a random intercept for participant to account for within-subject correlation across repeated measures. An unstructured covariance matrix is specified, with model fit compared against compound symmetry using the Akaike Information Criterion. The assumption of normally distributed residuals will be assessed using Q-Q plots and the Shapiro–Wilk test; non-normal outcomes will be analysed using generalized linear mixed models with appropriate link functions. Multiple comparisons across secondary outcomes are controlled using the Benjamini-Hochberg False Discovery Rate procedure at q = 0.05.

Exploratory subgroup analyses examine treatment effect heterogeneity by stroke chronicity (<6 months vs. ≥6 months), baseline severity (FMA-UE < 30 vs. ≥30), age (<65 vs. ≥65 years), and cognitive status (MoCA 21–25 vs. >25) using interaction terms in the mixed models. These are hypothesis-generating and interpreted cautiously given the sample size.

The economic evaluation calculates incremental cost-effectiveness ratios (ICERs) comparing total costs of the TRUST intervention against usual care (historical cost data), with effectiveness expressed as quality-adjusted life years (QALYs) derived from EQ-5D-5L utility scores. A 12-month Markov model with three health states (independent, dependent, deceased) is constructed, with transition probabilities informed by observed clinical trajectories and published literature. Costs and QALYs are discounted at 3% per annum. Uncertainty is characterised through probabilistic sensitivity analysis using 10,000 Monte Carlo simulations with parameters drawn from appropriate distributions (gamma for costs, beta for utilities, log-normal for relative effects), presented as cost-effectiveness acceptability curves against Singapore-specific willingness-to-pay thresholds. Missing data are handled using multiple imputation by chained equations under the missing-at-random assumption, with sensitivity analyses under missing-not-at-random scenarios.

### 2.8 Ethical considerations

The study received approval from National Healthcare Group Domain Specific Review Board (reference: 2023/00954) with full compliance to Singapore’s Human Biomedical Research Act and ICH-GCP guidelines. Informed consent emphasizes voluntary participation with forms translated into Chinese, Malay, and Tamil, requiring legally authorized representative consent for cognitive impairment (MOCA 21-25) with ongoing assent assessment.

## 3 Discussion

This pragmatic trial directly addresses critical barriers that have prevented robot-aided therapy translation despite decades of efficacy evidence. By evaluating home-based delivery using affordable, user-friendly, adaptive technology, we test a fundamentally different implementation model that could democratize access to intensive rehabilitation. The multi-site design across diverse healthcare settings provides natural variation in patient demographics and impairment characteristics, clinical workflows, cost structures and organizational cultures, enhancing confidence that successful results could translate broadly across Singapore’s healthcare system and potentially to similar developed Asian and international contexts. The emphasis on implementation outcomes alongside clinical effectiveness recognizes that pristine efficacy data from controlled trials often fails to predict real-world success, with much evidence-based interventions failing during implementation due to inadequate attention to contextual factors, stakeholder engagement, and sustainability planning.

The combinatory (home-RAT and clinic COT) service delivery model combining home-RAT with scheduled clinic visits and periodic outcome evaluation represents innovation in rehabilitation care architecture. Far from replacing therapists with robots, this approach upskills and repositions clinical rehabilitation professionals as conductors, remotely orchestrating technology-enabled home programs while reserving direct contact for high-value interventions requiring hands-on techniques to translate RAT gains to functional improvements. This could substantially increase therapist’s capacity to serve more patients while maintaining or potentially improving care quality through continuous objective monitoring and data-driven decision making. Furthermore, productivity gains could extrapolate to home situations as only carers were needed to supervise participants if needed during home-RAT.

Comprehensive economic evaluation provides crucial evidence for policy decisions in resource-constrained health systems facing a growing stroke burden. If home-RAT demonstrates comparable clinical effectiveness at lower total societal cost, the value proposition becomes compelling for health system transformation. The inclusion of willingness-to-pay assessment captures patient preferences and perceived value often overlooked in technology adoption decisions but critical for sustainable implementation and appropriate pricing strategies. Success could catalyze multiple policy changes including development of regulatory frameworks for home-based rehabilitation technology, quality standards for remote monitoring and supervision, reimbursement models covering technology-enabled care.

Several limitations warrant consideration when interpreting results. The single-arm design, while pragmatic and ethically justified given existing robust evidence, limits causal inference compared to randomized controlled trials, though comparison with robust historical controls and sensitivity analyses partially address this limitation. The 24-week follow-up may miss important long-term outcomes occurring years after stroke, including durability of gains, and technology abandonment, though cloud performance tracking. Technology requirements including basic internet connectivity and smartphone access may exclude digitally disadvantaged populations, potentially exacerbating health disparities despite efforts to minimize technical barriers through simplified interfaces and comprehensive training. The requirement for stable home environments and available caregivers may exclude vulnerable populations including those living alone or in institutional care, limiting generalizability to those potentially most needing innovative rehabilitation solutions.

This study establishes foundations for multiple future research directions including dose–response optimization studies to individualize therapy parameters, predictive analytics using machine learning to identify optimal patient selection criteria, technology enhancements incorporating artificial intelligence for adaptive difficulty adjustment and automated assessment, and population expansion to other neurological conditions. Implementation science investigations will develop and test strategies for scaling successful models across diverse healthcare contexts, while international collaboration could adapt the approach for low- and middle-income countries where rehabilitation services remain severely limited.

### 3.1 Risk mitigation measures

These include progressive home-RAT training protocols with 2-h daily limits, 24/7 research assistant/therapist/technical support with phone advice and replacement devices, multilayered data encryption, and quarterly safety monitoring by an independent committee with remote and phone/messaging advice and replacement pre-specified stopping rules for adverse events >10%, dropout >25%, or technical failures >15%.

## 4 Conclusion

The TRUST study represents a much needed and critical investigation at the convergence of rehabilitation science and clinical practice, implementation research, and health system innovation, addressing fundamental questions about the feasibility, acceptability, and cost effectiveness of home-RAT for stroke rehabilitation. By evaluating real-world implementation across diverse clinical settings, this research provides comprehensive evidence needed to inform practice transformation and policy development in an era of demographic transition, technological advancement, and evolving healthcare delivery models. Success would demonstrate that intensive, evidence-based rehabilitation can be delivered effectively in patients’ homes using affordable technology with remote clinical oversight, potentially solving the post-acute rehabilitation gap that compromises recovery for millions of stroke survivors globally.

The study’s comprehensive approach encompassing clinical outcomes, economic evaluation, technology acceptance, and implementation factors provides the multifaceted evidence required for complex health system interventions. Beyond immediate clinical implications, findings will contribute to broader understanding of how healthcare systems can leverage technology to address workforce limitations, demographic pressures, and patient preferences while maintaining or improving care quality and equity. The convergence of portable robotics, telehealth infrastructure, and implementation science creates unprecedented opportunities to transform rehabilitation delivery rehabilitation outcomes: telehealth and positions Singapore as a leader in telerehabilitation robotics.
